# Temporal Variation of Streamflow, Sediment Load and Their Relationship in the Yellow River Basin, China

**DOI:** 10.1371/journal.pone.0091048

**Published:** 2014-03-06

**Authors:** Guangju Zhao, Xingmin Mu, Alex Strehmel, Peng Tian

**Affiliations:** 1 Institute of Soil and Water Conservation, Northwest A&F University, Yangling, Shaanxi Province, China; 2 Institute of Soil and Water Conservation, Chinese Academy of Sciences & Ministry of Water Resources, Yangling, Shaanxi Province, China; 3 University of Kiel, Institute of Natural Resource Conservation, Department of Hydrology and Water Resources Management, Kiel, Germany; 4 College of Resource and Environment, Northwest A&F University, Yangling, Shaanxi Province, China; University of Aveiro, Portugal

## Abstract

Variation of streamflow and sediment load in the Yellow River basin has received considerable attention due to its drastic reduction during the past several decades. This paper presents a detailed investigation on the changes of streamflow and sediment load from 1952 to 2011 using monthly observations at four gauging stations along the Yellow River. The results show significant decreasing trends for both streamflow and sediment load at all four gauging stations over the past 60 years. The wavelet transform demonstrated discontinuous periodicities from 1969 to 1973 and after 1986 due to the construction of large reservoirs and implementation of numerous soil and water conservations practices. The sediment rating curves with the power-law function was applied to investigate the relationship between discharge and sediment load. The results indicate distinct variations of the relationship between streamflow and sediment and implied significant hydro-morphological changes within different periods. The reducing sediment supply from the source region and the increased erosive power of the river are detected at Lanzhou station, while the decrease of the transport capacity at Toudaoguai is caused by severe siltation. Significant changes in the relationship between streamflow and sediment load are found at Huayuankou and Gaocun stations, which are largely induced by evident sediment income and trapping effects of large reservoirs. It is estimated that numerous reservoirs have strongly altered the regime and magnitude of streamflow and trapped large amount of sediment, leading to severe siltation and evident reduction of their total volumes. A decrease in precipitation, incoming water from the upper reaches, soil and water conservation measures as well as water consumption contribute most to the significant reduction of streamflow. The decrease of sediment load mainly resulted from various soil and water conservation measures and trapping in reservoirs from 1986 to 2011.

## Introduction

Rivers are considered as the major link between continents and oceans and play a critical role in geological, biological and chemical processes on the land surface [1], [2]. Water discharge and sediment transport in the rivers greatly affect the geomorphology of the river channels, alluvial plains and deltas, and deliver numerous terrestrial materials to oceans, which sustain the coastal and marine ecosystems [3]. During the past century, river systems encountered significant changes at a global scale due to climate change and intensive human activities (land use change, river regulations, water abstraction, damming, sand mining etc.) [2], [4], [5]. As a result, a decrease or increase in streamflow and sediment load in many of the rivers throughout the world can be observed. Thus, it is of great scientific and practical importance to understand the changes of river systems and evaluate their possible controlling factors. Quantitative analysis on the spatial and temporal variation of streamflow and sediment load can provide a good reference for flood mitigation, river channel training and river basin management in the future.

In recent decades, variation in streamflow and sediment load has attracted considerable attention since human activities have greatly influenced the river systems worldwide [6]–[9]. These studies have reported that many large rivers throughout the world (i.e. Nile River, Colorado River, Amazon River, Yangtze River and Yellow River) show a significant decline in sediment load following the construction of reservoirs and dams and land use/cover changes [1], [10]–[12]. Walling and Fang (2003) addressed that statistically significant increasing and decreasing trends were both detected in approximately half of the 145 major rivers’ long-term sediment records that they examined [2]. Syvitski et al. (2005) computed the flux of terrestrial sediment to the oceans under contemporary and pre-human conditions, and predicted a global suspended sediment yield of 14.0×10^9^ t/a under pristine level without human activities, reducing to 12.6×10^9^ t/a for the anthropogenic phase [6]. Furthermore, the estimation indicated that on a global scale 26% of the sediment that would be transported to the coast and to deltas has been intercepted by retention in reservoirs.

The Yellow River is the second largest river in China, and is regarded as the “cradle of the Chinese civilization”. The Yellow River plays a critical role in the development of regional economy as the major source of freshwater for about 1.07×10^8^ people in the river basin. The total surface water consumption within the whole basin has increased from 12.9 km^3^ during 1950s to 18.2 km^3^ between 2000 and 2010 [7], [10]. Since the 1950s, the water discharge in the Yellow River has decreased due to impacts of climate changes and anthropogenic influences coupled with the harsh natural conditions and fragile ecosystem [13]–[17]. In the recent ten years (2001–2010), the average annual streamflow decreased to 242.6 ×10^8^ m^3^/a, account for only 53% of the discharge observed in the 1950s. Similarly, sediment load showed a synchronous decline with the dramatic changes in streamflow. The mean observed annual sediment load from 2001 to 2010 at Huayuankou station was 1.42×10^8^ t/a, only 9.7% of in the load in the 1950s.

Because of its important geographical, cultural and environmental functions and great decline of water resources and sediment flux to the sea, numerous studies have been conducted to quantitatively assess the changes in streamflow and sediment load as well as their potential causes [7], [10], [18]. Zheng et al. (2009) reported that land uses changes were predominant factors for the reduction in mean annual streamflow in the headwater catchment of the Yellow River basin [19]. Gao et al. (2011) investigated the changes of streamflow and sediment load in the middle reaches of the Yellow River, and found that the decreasing precipitation accounted for 28% of the reduction in streamflow from 1986 to 2008, and the remaining 72% is resulted from intensive human activities [20]. Peng et al. (2010) conducted a detailed assessment on the variation of sediment load in the upper, middle and lower reaches of the Yellow River during the past 60 years [21]. The results indicated that the construction of reservoirs and soil-water conservation measures were the dominant factors, contributing approximately 80% of sediment reduction, and the remainder was attributed to decreasing precipitation.

Many studies have been conducted on the changes of water discharge and sediment load at different spatial scales in the Yellow River basin. However, the studies were mostly conducted by making use of average annual records [15], [20]. Few studies were undertaken at a monthly scale with continuously updated data. Furthermore, very few studies have been conducted to address the relationship between streamflow and sediment load and the hydro-morphological responses to the changes of water discharge and sediment load in the Yellow River basin. In addition, the “Grain for Green” Program (GFGP) and the “Water-Sediment Regulation Project” (WSRP) were launched by the Chinese government in 1999 and 2002, respectively. The GFGP was intended to conserve the slope lands and effectively control soil erosion from the sources, while the WSRP aimed to scour large amounts of sediment, which are deposited in the reservoirs and channels in the middle and lower reaches. Until now, the changing properties of streamflow and sediment load have not been extensively addressed at the monthly scale, particularly after the operation of the GFGP and the WSRP. The objectives of this study are to investigate the changes in monthly streamflow and sediment load regime and their relationship using long-term observations along the Yellow River. In connection to that, potential influencing factors such as dams construction, soil and water conservation measures and other human activities are analyzed.

## Study Area and Data

**Figure 1:**
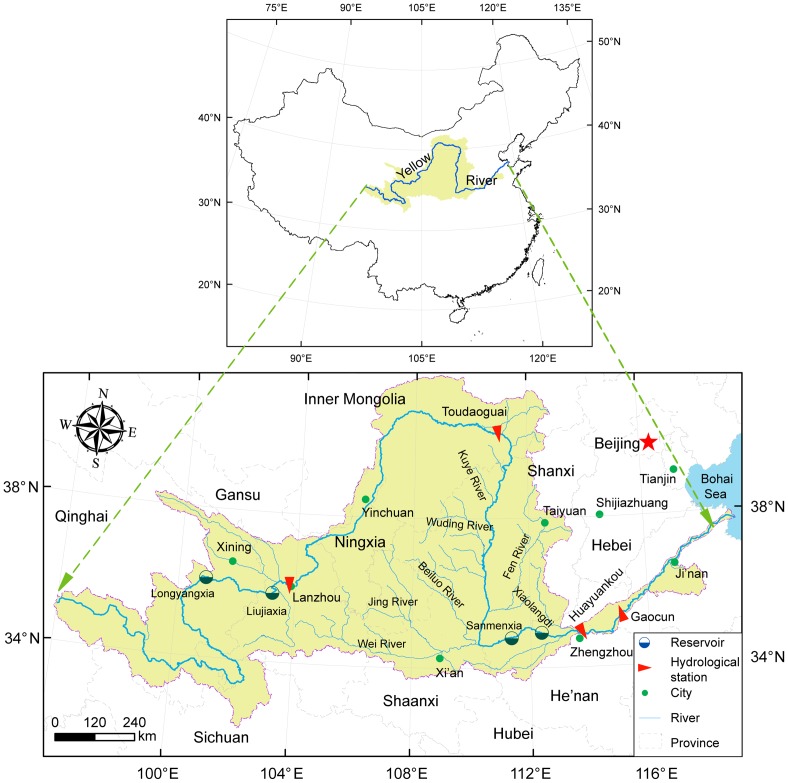
Location of the Yellow River basin, its major tributaries, key gauging stations and reservoirs.

The Yellow River originates from the Qinghai-Tibetan Plateau in western China, and flows eastward through the Loess Plateau and the North China Plain with a total length of 5,464 km (Fig. 1). The whole river basin covers approximately 75.2×10^4^ km^2^ and drains into the Bohai Sea. The river can be divided into three segments. The upper reaches extend over a length of 3,471 km from the river source to the Toudaoguai gauging station covering an area of 38.6×10^4^ km^2^, flowing through a mountainous region to the fluvial Hetao plain. The middle reaches are located between Toudaoguai and Huayuankou, and flows over 1,206 km through the highly erodible Loess Plateau with an area of 34.4×10^4^ km^2^. The lower reaches stretch from Huayuankou to the river mouth through a relatively flat alluvial plain with a drainage area of 2.3×10^4^ km^2^ (Fig. 1).

**Figure 2:**
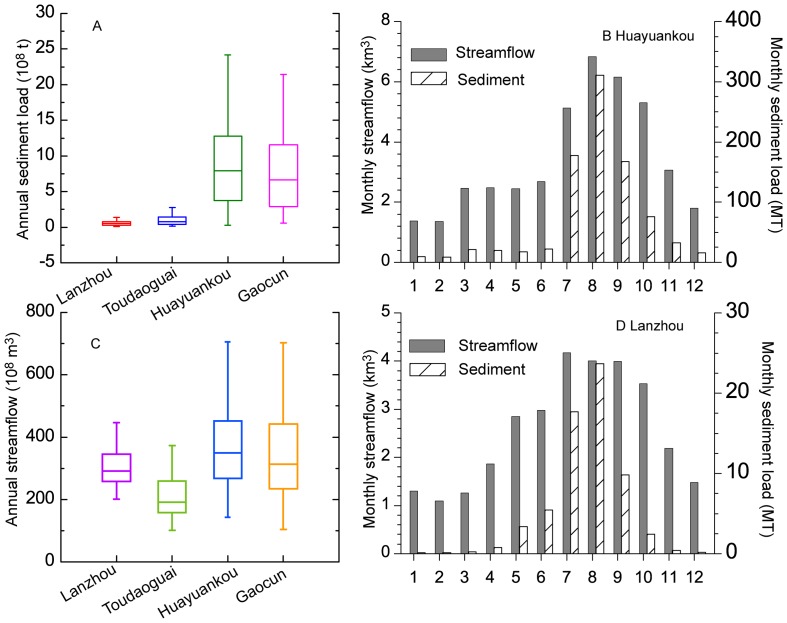
Streamflow and sediment load at four gauging stations along the Yellow River.

The average annual streamflow from the upper and middle reaches recorded at Lanzhou and Toudaoguai stations are 30.7 km^3^/a and 21.2 km^3^/a, respectively, and relatively lower than those at Huayuankou and Gaocun station in the downstream reach. While average annual sediment load amounts to nearly 0.64 ×10^8^ t/a at Toudaoguai station, and is much lower than that at Huayuankou station. More than 30 tributaries with a catchment area larger than 1,000 km^2^ (including Huangfu River, Kuye River, Tuwei River, Wuding River, Yan River, Wei River and Fen River and others) contribute approximately 80% of the sediment to the Yellow River [22]. The lower reach has become a narrow corridor and “suspended” river segment since the dominant deposition process of sediment led to the raising of the channel bottom above the ground level, sometimes by more than 10 m [23]. As a result, the mean annual sediment load at Huayuankou station averages to 8.79×10^8^ t/a, and is higher than the measurements at the downstream stations (i.e. Gaocun and Lijin stations). Accordingly, the sediment load at all four gauging stations along the mainstream indicates that the sediment is predominantly derived from the middle reaches of the Yellow River (Fig. 2a), where severe soil erosion occurs in the gullies and inter-gullies region [24]. However, the water from the middle reaches accounts for less than 50% of the discharge to the Bohai Sea (Table 1, Fig. 2c).

**Table 1:** General characteristics of the four gauging stations along the Yellow River.

Stations	Length of segment (km)	Controlled area (10^4^ km^2^)	Period	Mean annual discharge (10^8^ m^3^/a)	Mean annual sediment (10^8^ t/a)
Lanzhou	2119	22.26	1952–2011	307.01	0.64
Toudaoguai	1352	38.6	1952–2011	212.11	1.01
Huayuankou	1206	73.00	1952–2011	373.09	8.79
Gaocun	185	73.41	1952–2011	347.73	7.92

The majority of the Yellow River basin is influenced by the temperate continental climate. The upper and lower reaches are influenced by the southwest Indian monsoon and southeast Asian summer monsoon, which control the spatial and temporal variability of precipitation in the river basin. The summer monsoon normally starts to influence the river basin in June and retreats in October, accounting for more than 60% of annual total rainfall. Streamflow is relatively higher during the rainy season, particularly from July to October. In contrast, high sediment loads are observed in the period between July and September due to frequently occurring rainstorms. The seasonal distributions of sediment load at the four gauging stations show a strong association with the patterns of water discharge, as characterized by the co-occurrence of high sediment loads and high water discharge during the rainy season (Fig. 2b, d).

The consecutive monthly data of streamflow and sediment yields at Lanzhou, Toudaoguai, Huayuankou and Gaocun stations from 1952 to 2011 were provided by the Yellow River Water Resources Commission (Table 1), and were partly collected from the Yellow River Sediment Bulletin [25]. The daily discharge was computed from the water level by using previously calibrated discharge-water level curves. Water was sampled at fixed intervals, and suspended sediment concentration was obtained by measuring water samples in the laboratory. All the measurements of water level, discharge, SSC followed national standards issued by the Ministry of Water Conservancy, and were printed in the Hydrological Year-book of the Yellow River. The monthly and annual streamflow and sediment load at the gauging stations were derived from the daily measured data. The accuracy and consistency of all the data used in this study have been checked out by the corresponding agencies before their release.

## Methodology

In this study, we applied a simple linear regression to detect the temporal trends of annual streamflow and sediment load, and significances were detected by F-test at p<0.05. The discrete wavelet transform and flow duration cure method were respectively used to identify the periodical characteristics and changes of hydrological regime for monthly time series. Additionally, flow-sediment relationship at four main gauges was examined by using sediment rating curve.

### Wavelet Analysis for Changes in the Streamflow and Sediment Regime

The wavelet transform is a spectral method of decomposing a time series into time and frequency, and is used to identify and analyze the dominant localized variation of power. The wavelet analysis has been widely applied to characterize the frequency, intensity, time position and duration of the variations for hydro-climatic time series [9], [26]. Given the time series of 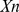, with equal time interval 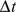 (one month in our study), a corresponding Morlet wavelet function 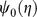, with a mean value of zero and localized in time and frequency space is defined as:



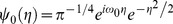
(1) Where 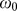 denotes the dimensionless frequency, and 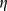 is the non-dimensional time frequency [27], [28]. The continuous wavelet transform of a discrete sequence 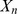 is defined as the convolution of 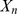 with a scaled and translated version of 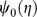:
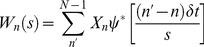
(2)Where the * represents the complex conjugate, 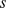 is the wavelet scale, and 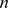 is the translated time index.

The statistical significance of wavelet spectra can be assessed under the null hypothesis that the signal is generated by a stationary process given the background power spectrum (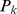). It is assumed that the time series has a mean power spectrum:
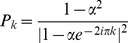
(3)where 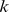 is the Fourier frequency index that corresponds to the wavelet scale 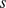. A specific confidence level against red noise, such as 95% confidence level (significance at 5%) can be obtained by [28]:
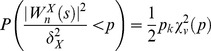
(4)where 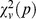 represents the value of Chi-square distribution with two degree of freedom at the confidence level 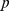, and 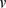 is equal to 1 for real and 2 for complex wavelet. The geophysical series have the red noise characteristics which can be modeled by a univariate lag-1 autoregressive (AR-1) process. A detailed explanation for the concept and procedure of the wavelet transform has been presented by previous studies [27], [28].

### Sediment Rating Curves

The sediment rating curve is defined as the statistical relationship between sediment discharge (*Qs*) or concentration (*Cs*) and water discharge (*Q*). It generally represents a functional relationship of the form [29], [30]:
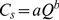
(5)




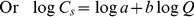
(6)in which *a* and *b* are the coefficients of the sediment rating curve, and their values can be obtained from the measurement data through a linear regression between log*Cs* and log*Q*. The sediment rating curve is regarded as a ‘black box’ type of model expressing the relationship between sediment and discharge as a power function. In general, the parameters *a* and *b* in the sediment rating curve do not contain particular physical meaning [31]. However, Morgan (1995) argued that the sediment rating parameter *a* was as an index of erosion severity in the river channel [32]. A high value of *a* is associated with the area, which is characterized by easily erodible materials and high loads of transported materials. The coefficient *b* depicts the erosive power of the river, with high values indicating strong increase in erosive power of the river. It has also been addressed that the coefficient *b* is related to the regional climate pattern, channel morphology, grain-size distribution of sediment and the erodibility within the river basin [31], [33].

### Flow Duration Curve

The flow duration curve (FDC) is a cumulative distribution function that shows the percentage of specific discharge that is equaled or exceeded. It has been considered as an important approach for assessing the frequency and magnitude of a time series. For a given time series 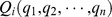, it can be ranked to produce an ordered record 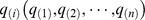, where *n* is the sample size, and 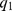 and 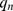 are the largest and smallest observations, respectively. The FDC can be plotted by the measured values 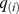 associated with probability of 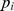, expressed as:
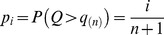
(7)


The FDC provides a simple graphical view of the overall variability for long term streamflow or sediment load [34]. It is probably one of the most informative approaches of displaying the complete range of hydrological time series.

## Results

### Trends Analysis for Streamflow and Sediment Load

**Figure 3:**
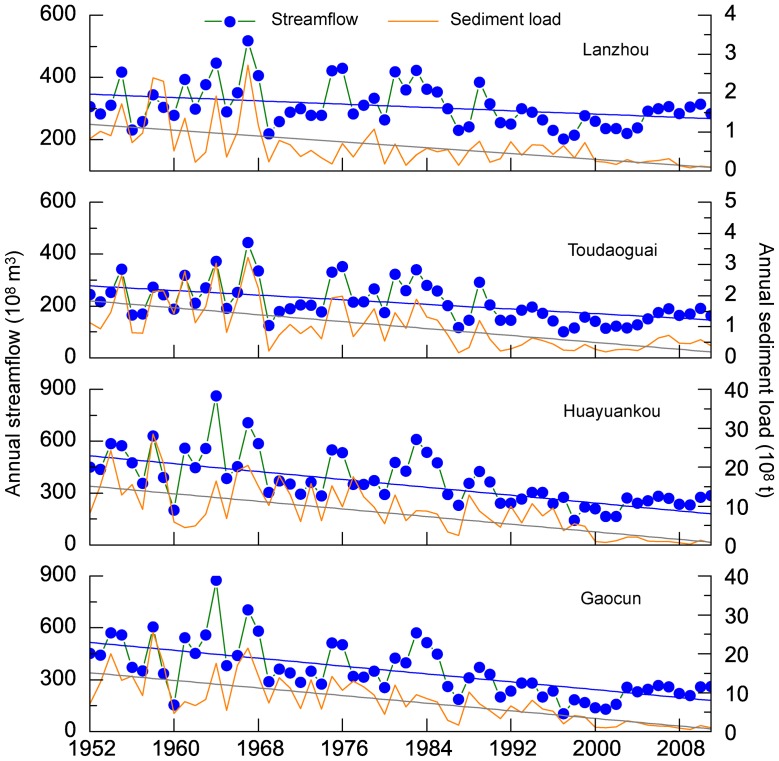
Trend analysis for annual runoff and sediment load in the Yellow River basin.

Fig. 3 shows simple linear trends in average annual streamflow and sediment load at Lanzhou, Toudaoguai and Huayuankou stations. Overall, both streamflow and sediment load shows significant decreasing trends (p<0.05) at all stations. The annual streamflow and sediment load exhibits the most significant decrease at Huayuankou station (p<0.05), with average reduction rates of −5.44×10^8^ m^3^/a and −0.26×10^8^ t/a, respectively, while the streamflow and sediment load at Lanzhou station display gently decrease (−1.33×10^8^ m^3^/a and −0.02×10^8^ t/a).

### Periodicities in Monthly Streamflow and Sediment Load

**Figure 4:**
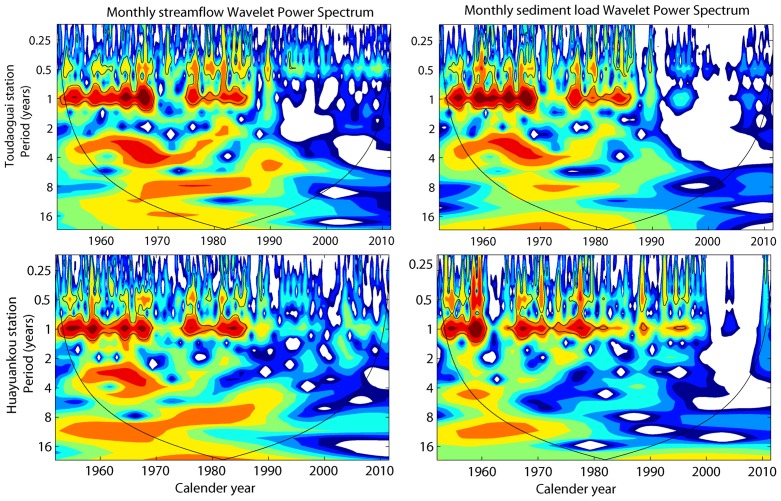
Wavelet analysis of monthly runoff and sediment load at Huayuankou stations.

The wavelet transform was applied to examine the variation of periodicities in monthly streamflow and sediment load at the four gauging stations. Fig. 4 illustrates the wavelet power spectrum of the monthly hydrological variables. It can be clearly seen that both streamflow and sediment load are in good agreement between low- and high-frequency oscillation. The wavelet power spectrums indicate intermittent periodicities of 0.5 and 1 year due to the seasonal and annual alternation of hydrological variables at all the stations. Furthermore, the 95% confidence intervals are continuously distributed in one year band during 1952–1968 and 1974–1986. Periodicity was not detected from 1969 to 1973 and after 1987. This can be attributed to the intensive human activities in the upper and middle reaches of the Yellow River during the past six decades. The data in Fig. 4 indicate that human activities have made the seasonal and annual cycles of monthly series weaker since the late 1980s, and even disappearing completely after 2000.

According to the wavelet transform results of monthly streamflow and sediment load, five periods can be distinguished to quantify the temporal changes of discharge and sediment in the Yellow River basin. Table 2 demonstrates the mean water discharge and sediment concentration at four gauging stations along the Yellow River during different periods. A general decrease in both water discharge and sediment concentration can be clearly detected.

**Table 2:** Mean water discharge (Q) and sediment concentration (Cs) at four gauging stations along the Yellow River within different periods.

Stations	Lanzhou		Toudaoguai		Huayuankou		Gaocun	
	Q (m^3^/s)	Cs (g/l)	Q (m^3^/s)	Cs (g/l)	Q (m^3^/s)	Cs (g/l))	Q (m^3^/s)	Cs (g/l))
1952–1968	1084.64	3.84	837.98	5.64	1614.25	43.98	1562.07	41.55
1969–1974	857.43	1.54	570.22	2.36	1038.76	36.55	1002.96	30.32
1975–1986	1125.14	1.61	849.66	4.06	1390.41	31.20	1286.34	29.05
1987–1999	842.01	1.61	516.63	1.41	880.97	22.55	753.55	16.66
2000–2011	865.06	0.63	481.37	1.31	764.31	3.20	696.06	4.47

Due to the length of the river basin, changing trends of mean water discharge and sediment concentration are highly variable, both spatially and temporally. In detail, the mean water discharge at Lanzhou station shows a reduction of nearly 20%, while the sediment concentration shows an evident decline from the period of 1952–1968 (3.84 g/l) to 2000–2011 (0.63 g/l). Compared to the Lanzhou station upstream, the mean water discharge decreased by nearly 50% or even more at the stations in the middle and lower reaches. A much more evident reduction (decreased by approximately 90%, Table 2) is observed for the sediment concentration at Huayuankou and Gaocun stations. The average annual sediment concentration during 2000–2011 was 3.2 g/l, only accounting for 7.3% of that from 1952 to 1968 at Huayuankou station.

### Sediment Rating Curves at Four Key Stations Along the Yellow River

**Figure 5:**
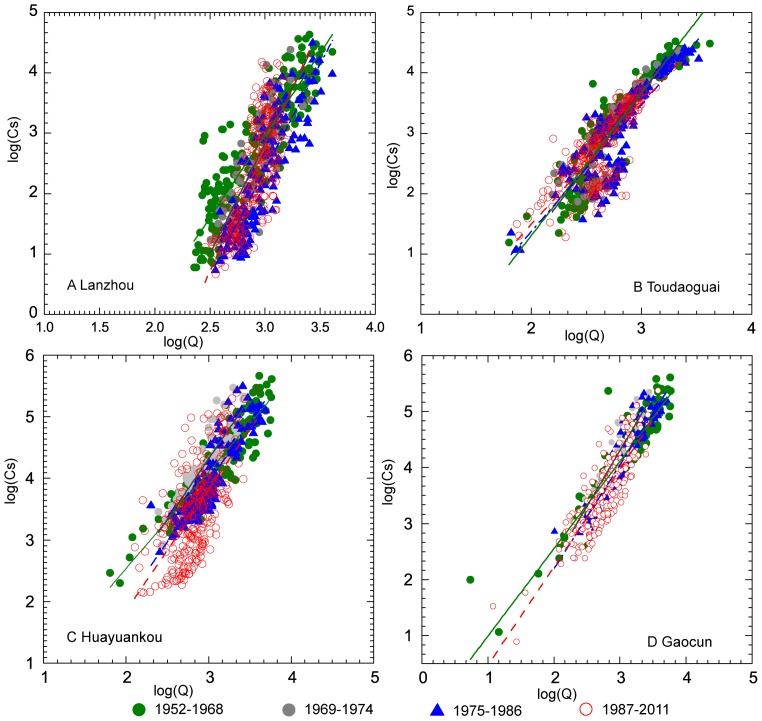
Sediment rating curve analysis at four gauging station along the Yellow River.

Sediment rating curves at four main gauging stations along the Yellow River were obtained for four distinct periods (i.e. 1952–1968, 1969–1974, 1975–1986, and 1987–2011) according to the periodicities of monthly streamflow and sediment load. All sediment rating curves at four stations in different periods generally displayed a decreasing trend in the later period (1987–2011) (Fig. 5). The steepest rating curves were found at Lanzhou, followed by Toudaoguai station, and the relatively flat rating curves were found at Huayuankou and Gaocun stations. This pattern may relate to the channel morphology of the rivers. High slope gradient and bedrock-confined V-shaped channels in the upstream of Lanzhou suggests higher unit stream power with steep rating curve. In contrast, the wide U-shape river channel in the mid-lower reaches is characterized by gentle slope gradient with lower unit stream power.

It has been mentioned that the coefficient log(a) represents the soil erodibility in the river basin. High values of log(a) denote high sediment supply. The variation of *a* with time for a given basin is expected to reflect the human disturbances on the hydrological processes. The exponent *b* represents the erosive power of the river and the influence on the sediment supply from the entire basin surface. Large values of *b* indicate an increased transport capacity of the river [31]. The decreasing log(a) and increasing *b* from 1952 to 2011 imply a decreasing sediment supply from the source region and an increased erosive power of the river at Lanzhou station (Table 3). However, the sediment rating exponent log(a) values at Toudaoguao station continuously increased from −3.44 in 1952–1968 to −2.52 in 1987–2011, and the rating coefficient *b* synchronously decreased from 2.37 to 2.01, suggesting increasing sediment supply and decreased transport capacity of the river due to severe siltation. The sediment rating parameters show similar changes at Huayuankou and Gaocun stations within the past six decades. Relatively high values of log(a) in 1952–1968 are induced by the abundant sediment income from the upper and middle reaches, whereas the higher values of *b* at lower reaches of Huayuankou and Gaocun from 1969 to 2011 might indicate the influence of the construction of the Sanmenxia Reservoir.

**Table 3:** Sediment rating parameters for different periods at four gauging stations.

Stations	Lanzhou			Toudaoguai			Huayuankou			Gaocun		
	log(a)	b	R^2^	log(a)	b	R^2^	log(a)	b	R^2^	log(a)	b	R^2^
1952–1968	−5.22	2.73	0.78	−3.44	2.37	0.86	−0.6	1.57	0.86	−0.57	1.56	0.88
1969–1974	−8.18	3.67	0.70	−3.60	2.42	0.68	−1.57	1.96	0.77	−1.69	1.99	0.88
1975–1986	−8.15	3.51	0.73	−2.83	2.10	0.75	−1.87	1.94	0.81	−1.52	1.86	0.88
1987–2011	−9.43	4.06	0.60	−2.52	2.01	0.60	−2.08	1.96	0.44	−1.29	1.75	0.71

### Flow Duration Curve Analysis for Monthly Streamflow and Sediment Load

**Figure 6:**
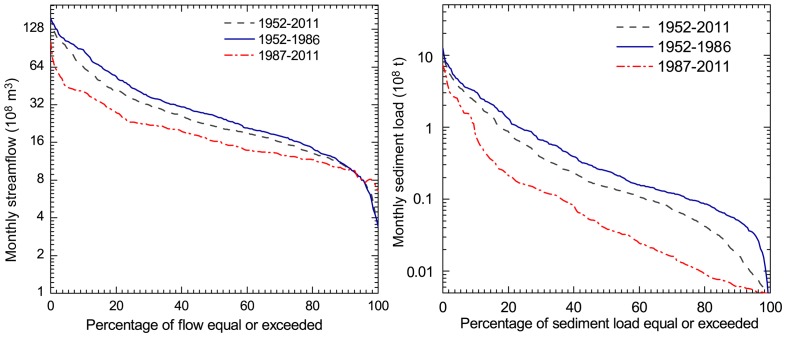
Flow duration curves for monthly runoff and sediment load at Huayuankou station.

According to the wavelet analysis of monthly streamflow and sediment load, we divided the studied period into two periods, namely before and after 1986, to simply quantify the variation in monthly streamflow and sediment load. Fig. 6 shows an illustrative example of the FDCs for both monthly streamflow and sediment load at Huayuankou station during the two periods described previously. The results indicate that there was evident decline for both mean monthly streamflow and sediment load. More than 50% of the reduction was detected when comparing the high values of flow and sediment load during 1987–2011 to those of 1952–1986.

To clearly identify the temporal variation of streamflow and sediment load at the four stations along the Yellow River, six indices with different magnitudes of flow and sediment load (i.e. Q*_10_*, Q*_50_*, Q*_90_*, Sed*_10_*, Sed*_15_*, and Sed*_90_*) were selected for analysis during two different periods. The indices of high-flow (Q*_10_*), median-flow (Q*_50_*) and low-flow (Q*_90_*) were defined as the specific values exceeded 10%, 50% and 90% of the monthly streamflow respectively. Similar indices were also defined for sediment load. As shown in Table 4, the magnitude of monthly streamflow and sediment load generally decreased during the period of 1987–2011 compared with that from 1952 to 1986 at all four key gauging stations. Specifically, the high-flow (Q*_10_*) reduced by more than 50% at all the stations except for the upstream Lanzhou station. However, the low-flow indicated relatively gentle changes between the two periods. In contrast, the high-sediment index (Sed*_10_*) within the period of 1987–2011 illustrated a drastic decline at the four stations, particularly at Toudaoguai, Huayuankou and Gaocun stations, where reductions of approximately 70% can be observed.

**Table 4:** Flow duration curve analysis for different periods at four gauging stations along the Yellow River.

Stations	Lanzhou		Toudaoguai		Huayuankou		Gaocun	
	Pre_1986_	Post_1986_	Pre_1986_	Post_1986_	Pre_1986_	Post_1986_	Pre_1986_	Post_1986_
Q*_10_* (10^8^m^3^)	56.25	32.92	45.26	22.50	88.13	40.44	81.96	36.69
Q*_50_* (10^8^m^3^)	22.79	22.01	14.93	10.55	26.18	16.37	24.19	14.49
Q*_90_* (10^8^m^3^)	12.24	11.89	6.22	6.51	10.45	9.75	7.53	8.81
Sed*_10_* (10^6^t)	20.5	8	39	9.86	308	76	274	70
Sed*_50_* (10^6^t)	0.67	0.66	354	177	25	4	26	6
Sed*_90_* (10^6^t)	0.04	0.04	25	30	5	1	4	2

## Discussion

### Impacts of Dam Operation on Streamflow and Sediment

Regulation from dams and reservoirs is the most direct way among the human activities that drastically alters the regimes of streamflow and sediment load in the river basin. In the Yellow River basin, more than 3,150 reservoirs have been built and the total storage capacity of all the registered reservoirs reaches up to 72 km^3^ [35]. This is much higher than the average annual streamflow of 24.4 km^3^ between 2001 and 2011 at Huayuankou station.

**Figure 7:**
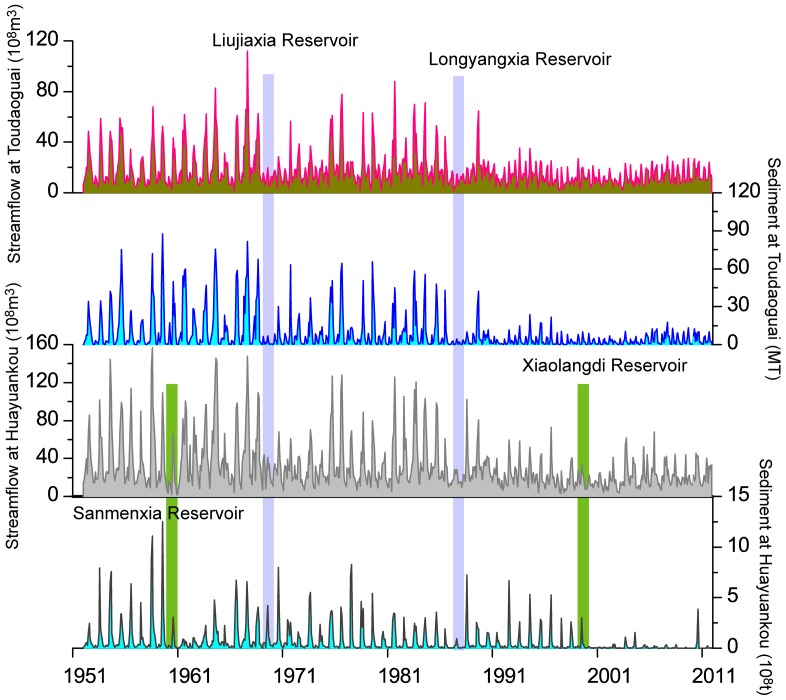
Monthly streamflow and sediment load at Toudaoguai and Huayuankou stations associated with reservoirs construction.

Fig. 7 illustrates the regulating effect of jointly operated reservoirs on the water and sediment discharge regimes at Toudaoguai and Huayuankou stations. The abrupt decline in monthly streamflow and sediment load indicates substantial trapping effects of four major reservoirs along the Yellow River: the Longyangxia (with a total volume of 27.6 km^3^), Liujiaxia (5.7 km^3^), Sanmenxia (9.6 km^3^) and Xiaolangdi Reservoir (12.7 km^3^). In general, the magnitude and variability of monthly streamflow at Toudaoguai and Huayuankou stations has been reduced by these large reservoirs, particularly after the late 1980s. The seasonal variability in both streamflow and sediment load has decreased since 1986 when the Longyangxia Reservoir started operation. The observed streamflow in the flood seasons (June to October) accounted for approximately 68.3% of the average annual total in the 1950s, but decreased to 53.1% after 2000 at Huayuankou station. This may be largely attributed to the trapping effect of reservoirs, which dramatically reduced the flood peaks by storing the water during the rainy season and releasing the water in the dry period to meet the agricultural demands. Furthermore, the discontinuous periodicities of monthly streamflow and sediment load (Fig. 4) were consistent with the timing of dams’ construction, particularly after the construction of the Longyangxia Reservoir in 1986.

**Figure 8:**
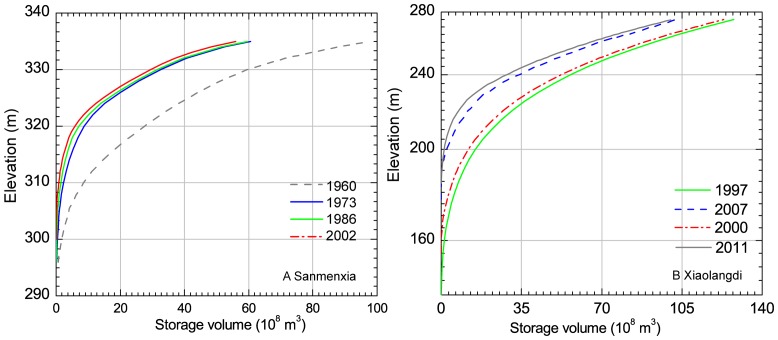
Historical changes of storage volumes for Sanmenxia and Xiaolangdi reservoirs.

The Sanmenxia reservoir started operation in 1960 and trapped large amounts of sediment during its first few years of operation due to abundant sediment discharged into the Yellow River from the Loess Plateau. As shown in Fig. 8a, the storage volume decreased significantly from 1960 to 1973, afterwards the reduction is relative gentle. Sediment retention was estimated to reach up to 79.1×10^8^ t with an average of 3.13×10^8^ t/a from 1960 to 1973 [25]. The sediment load at Huayuankou station was 11.06×10^8^ t/a during the period of 1960–1973, which has been reduced by nearly 35% compared to that from 1952 to 1959.

In order to reduce severe siltation in the lower reaches of the Yellow River, the Xiaolangdi reservoir was constructed between Sanmenxia reservoir and Huayuankou station (Fig. 1) and started to store water in 1999. Considerable siltation occurred with the operation of the reservoir, leading to a total sediment retention of about 32.5×10^8^ t from 1997 to 2007 (Fig. 8b). However, the construction of Xiaolangdi reservoir did not resolve the severe siltation in the lower reaches of the Yellow River. To efficiently mitigate siltation and scour the sediment deposited in the channel, the Yellow River Conservancy Commission initiated the Water-Sediment Regulation Scheme in 2002. This scheme aims at the joint operation of large reservoirs, which releases large amounts of stored water to deliver sediment retained within the Xiaolangdi reservoir downstream and to scour the lower reaches in the summer season. It has been estimated that about 3.9 ×10^8^ t of sediment have been scoured into the sea after executing this measure 13 times between 2002 to 2011 with a total released water volume of 50.91 km^3^ [25].

### Effects of Soil and Water Conservation on Streamflow and Sediment Load

A series of soil and water conservation practices have been carried out in the upper and middle reaches of the Yellow River basin since the late 1950s. These soil and water conservation measures include biological measures (i.e. terracing, afforestation) and engineering measures (such as check dams and reservoirs) [36]. Table 5 shows statistics of soil and water conservation measures in the middle reaches of the Yellow River basin. The measures present various increase rates during different periods. Before the late 1970s, the area controlled by soil and water conservation measures was very small due to the limited awareness and funding, and increased significantly afterwards because of considerable government-funded projects. Between the 1970s and 1996, the area controlled by various types of soil and water conservation practices has almost tripled and recent statistics indicate that nearly 0.2×10^6^ km^2^ of area are controlled with various soil and water conservation measures by 2006, including more than 1, 800 key hydraulic projects and 110,000 check dams for gully erosion control [37]. Thus, it can be undoubtedly concluded that the dramatically increased soil and water conservation measures are responsible for the decreasing streamflow and sediment load in those catchments. Generally, monthly streamflow and sediment load of the Yellow River demonstrated seasonal cycles before 1970s without intensively intervention from human activities. However, the discontinuous seasonal cycles on monthly streamflow and sediment load indicated that various soil and water conservation measures had greatly altered the hydrology regime of the river and reduced the discharge and sediment load (Fig. 4 and 6), particularly after the 1980s.

**Table 5:** Statistics of soil and water conservation measures in the middle reaches of the Yellow River basin [37], [39].

Soil-water conservation measures	Area controlled by various measures during different period (10^3^ km^2^)					
	1959	1969	1979	1989	1996	2006
Terrace	0.53	2.41	5.62	10.06	14.41	28.54
Afforestation	1.97	5.21	13.56	33.31	44.40	58.61
Grassing	0.42	0.66	1.57	5.51	6.37	14.07
Check dam	0.036	0.185	0.467	1.009	1.224	1.310
In Total	2.95	8.47	21.22	49.89	66.41	102.53

Associated with large number of soil and water conservation measures implementation, land use and land cover showed great changes in the whole river basin. Increasing cropland, built-up land and bare land have been detected from 1980s to 2000 according to Landsat TM interpretation, the areas of grassland and woodland decreased dramatically [16], [38]. Zheng et al. (2009) indicated that land use changes were responsible for more than 70% of the streamflow reduction in the 1990s [19]. Since 1999, vegetation cover exhibited an increasing trend due to the GFGP [17]. The increasing vegetation cover may result in more water remained in the soil store for evaporation and then reduce river runoff. Zhang et al. (2008) addressed that land use/cover changes (including soil and water conservation measures) accounted for over 50% of the reduction in mean annual streamflow in most studied catchments in the middle reaches of the Yellow River basin [36].

### Relative Contributions of Precipitation and Anthropological Effects

Consistent with previous studies [18], [40], both wavelet transform and FDC analysis suggested that the magnitude and regime of streamflow and sediment have been greatly altered, and a more uniform runoff and sediment regime has formed. It has also been confirmed that the significant decrease in streamflow and sediment load of the Yellow River resulted mainly from the variability in precipitation and anthropogenic activities [13], [14]. To quantitatively separate the contribution of precipitation and human activities on streamflow and sediment load reductions, numerous studies applied simple linear regression approaches or water balance-based methods [15], [18], [21]. In this study, we applied a simple linear regression method based on approximating the relationship between changes of streamflow/sediment and precipitation to estimate the impact of precipitation variability on them in the river basin.

**Figure 9:**
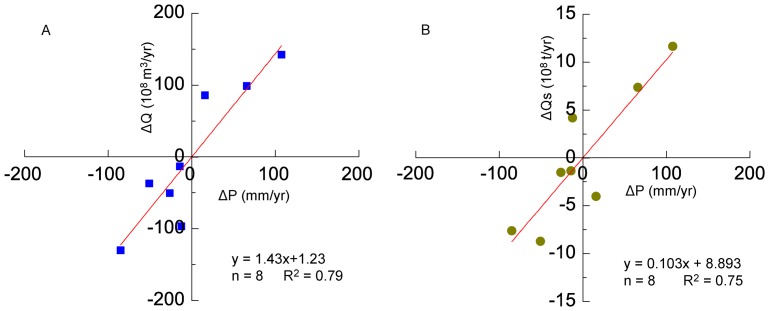
Relationship between the interannual variability of precipitation streamflow and sediment load at Huayuankou from 1952 to 1959.

Consistently to previous analysis and other studies [21], we assumed that streamflow and sediment load were not intensively influenced by human activities in the period of 1952 to 1959, which can therefore be considered as a reference period. Fig. 9 illustrates the relationship between the inter-annual variability of precipitation (ΔP) and the inter-annual variability of streamflow/sediment (ΔQ/ΔQs) at Huayuankou station. By developing the relationship shown in Fig. 9, contributions for precipitation and human activities to the decrease of streamflow and sediment load can be estimated (shown in Table 6).

**Table 6:** Relative contribution of precipitation and human activities to the decrease of streamflow and sediment load at Huayuankou within different periods.

	Streamflow (10^8^ m^3^/a)			Sediment load (10^8^ t/a)		
Time period	1960–1986	1987–1999	2000–2011	1960–1986	1987–1999	2000–2011
Average precipitation	490–469	490–448	490–443	490–469	490–448	490–443
average streamflow and sediment	487.4–443.8	487.4–277.8	487.4–241.0	16.8–10.7	16.8–7.1	16.8–1.0
Decrease in streamflow and sediment	43.6	209.6	246.4	6.1	9.7	15.8
	**Contribution to streamflow reduction (10^8^ m^3^/a)**			**Contribution to sediment load reduction (10^8^ t/a)**		
Soil-water conservation^a^	–	45.8	74.5	1.8	3.1	6.1
Trapping by Reservoirs^b^	6.4	2.1	2.4	3.1	1.5	3.99
Changes from upper reaches	–	73.2	84.3	0.10	1.06	1.10
Precipitation variation^c^	16.6	34.1	38.3	1.3	2.7	3.0
Changes in water consumption^b^	14.5	20.9	15.2	–	–	–
Total decrease	37.5	176.1	214.7	6.3	8.36	14.19

a Data was obtained from [41] and [42];

b data from YRCC [25];

c values are estimated from the equation shown in Fig. 9.

The average precipitation from 1960 to 1987 was 461 mm, 4 mm lower than the reference levels. The decrease of streamflow was estimated to be 0.29×10^8^ m^3^/a according to the relationship between ΔQ and ΔP, and as shown in the equation in Fig. 9a (Table 6). The changes of precipitation contributed approximately 1.3×10^8^ t/a to the sediment load reduction based on the equation displayed in Fig. 9b. During this period, the decreased streamflow and sediment trapped by reservoirs were 6.4×10^8^ m^3^/a and 3.1×10^8^ t/a, with a large portion of the reduction at Huayuankou station (Table 6). However, soil and water conservation started from the late 1950s and became effective since the late 1970s [18].

The average precipitation from 1987 to 1999 was 420 mm, 45 mm below that from 1952 to 1959. According to the equation shown in Fig. 9, this results in a reduction in streamflow of 34.1×10^8^ m^3^/a and sediment load of 3.7×10^8^ t/a. Dramatic decrease in streamflow was detected due to an evident decline of incoming water from the upper reaches and soil and water conservation measures in the middle reaches (Table 6). Sediment retention by reservoirs decreased to 1.5×10^8^ t/a, accounting for 15.5% of the measured decrease at Huayuankou station. Variation in precipitation and soil and water conservation measures were two predominant factors leading to 2.7×10^8^, and 3.1×10^8^ t/a of sediment decrease at Huayuankou station. In summary, the total decreases of streamflow and sediment load due to these factors were 176.1×10^8^ m^3^/a and 8.36×10^8^ t/a respectively, which were slightly lower than the measured decrease at Huayuankou station. This may be caused by the underestimation of water consumption and effects of soil and water conservation.

The average precipitation from 2000 to 2011 was 443 mm, which resulted in decrease of streamflow and sediment load 38.3×10^8^ m^3^/a and 3.0×10^8^ t/a. In this period, soil and water conservation practices became the dominant factor, accounting for 30.2% and 38.6% of the measured decrease in streamflow and sediment load at Huayuankou station. Large reservoirs built within this period caused an increasing trapping effect and a sediment decrease with an average rate of 3.99×10^8^ t/a. The total decrease of sediment load due to these factors was 14.29×10^8^ t/a, which agrees relatively well with the measured decline at Huayuankou station (Table 6).

## Conclusion

This study investigated the variation of monthly streamflow and sediment load at four key gauging stations (Lanzhou, Toudaoguai, Huayuankou and Gaocun) along the Yellow River from 1952 to 2011 by making use of the wavelet transform, sediment rating curve and flow duration curve methods. The findings can be summarized as follows:

Both streamflow and sediment load, demonstrate significant decreasing trends (p<0.05) at all the stations. The most significant decline in annual streamflow and sediment load is detected at Huayuankou station, with average reduction rates of −5.44×10^8^ m^3^/a and −0.26×10^8^ t/a, respectively, while the streamflow and sediment load at Lanzhou station display a gentle decrease (−1.33×10^8^ m^3^/a and −0.02×10^8^ t/a).

The wavelet transform revealed intermittent periodicities of 0.5 and 1 year within 95% confidence interval due to the seasonal and annual alternation of hydro-climatic variables. Periodicity was not detected from 1969 to 1973 and after 1986. This can be attributed to the intensive human activities in the upper and middle reaches of the Yellow River during the past six decades.

Sediment rating curves at four gauging stations generally displayed decreasing trends in response to the decline of sediment induced by human activities. The decreasing log(a) and increasing *b* imply a reduced sediment supply from the source region and an increase of the erosive power of the river at Lanzhou station. However, the Toudaoguai station showed an opposite trend with decreasing transport capacity due to severe siltation. Similar changes were found at Huayuankou and Gaocun stations. Relatively high values of log(a) in 1952–1968 were induced by the abundant sediment income from the upper and middle reaches, whereas the increasing *b* from 1969 to 2011 indicates trapping effects from large reservoirs.

Flow duration curve analysis showed that the regime and magnitude of monthly streamflow and sediment load have been completely altered from 1952 to 2011. Compared to the changes of streamflow, sediment indices illustrated a dramatic decline at the four stations, particularly in the mid- and lower reaches with reduction rates of more than 70%.

Significant reduction of streamflow and sediment load is largely caused by reservoir construction and the implementation of numerous soil and water conservation measures. Precipitation changes, incoming water from the upper reaches, water consumption and soil and water conservation measures are the dominant factors for the decrease in streamflow. Sediment retention by Sanmenxia and Xiaolangdi reservoirs is related to great trapping effects on sediment discharge since their operation, leading to an evident reduction in their total volumes. Variation in precipitation and various soil and water conservation practices also contribute a large proportion to the sediment reduction during 1986 to 2011.
